# The modified horn flap for nasal tip reconstruction

**DOI:** 10.1016/j.jpra.2021.03.006

**Published:** 2021-04-21

**Authors:** Parthena I. Deskoulidi, Russell Aldred

**Affiliations:** Department of Plastic and Reconstructive Surgery, St. Vincent's Hospital, 390 Victoria St, Darlinghurst, Sydney, NSW 2010, Australia

**Keywords:** Horn flap, Superior alar artery, Nasal tip reconstruction, Deckling incision

## Abstract

Numerous flap design techniques have been proposed for soft tissue reconstruction of the nasal tip.

The modified horn flap based on the superior alar artery and nasalis muscle is the preferred option for defects ranging in size from 1 to 2 cm vertically and 1.5 to 3 cm horizontally after skin cancer excision. This innovative technique is a reliable and versatile island flap for reconstruction of the nasal tip in a one-stage operation, providing successful functional and aesthetic results, as tissues for the flap are generated from the nose. The contribution of the superior alar artery, which forms the vascular axis of the flap, plays a vital role in flap survival – together with part of the nasalis muscle.

## Introduction

Soft tissue reconstruction of the nasal tip is a delicate art. The goal in nasal tip reconstruction after skin cancer excision is the restoration of symmetry, contour, color match and an overall pleasing result. The nasal tip is usually thick, inelastic, and often sebaceous. Numerous techniques^1^ have been utilized for nasal tip reconstruction – including skin grafts and local flaps – with their advantages and drawbacks. In 1992, O'Donnell et al.^2^ described the horn-shaped V–Y advancement flap as a reliable method following excision of small facial lesions, particularly in the medial canthal area. In 2014, Schopper et al.^3^ developed a single-pedicled nasalis musculocutaneous flap to repair defects of the distal nose. In 1985 Marchac and Toth^4^ described the axial-frontonasal flap based on a pedicle which is a branch of the angular artery, joining with supraorbital arteries. In 1993 de Fontaine et al.^5^ further defined this flap with the pedicle on the same side as the lesion.

The modified horn flap based on the superior alar artery as well as the nasalis muscle is the preferred option for the authors.

## Surgical technique

**Figure 1:**
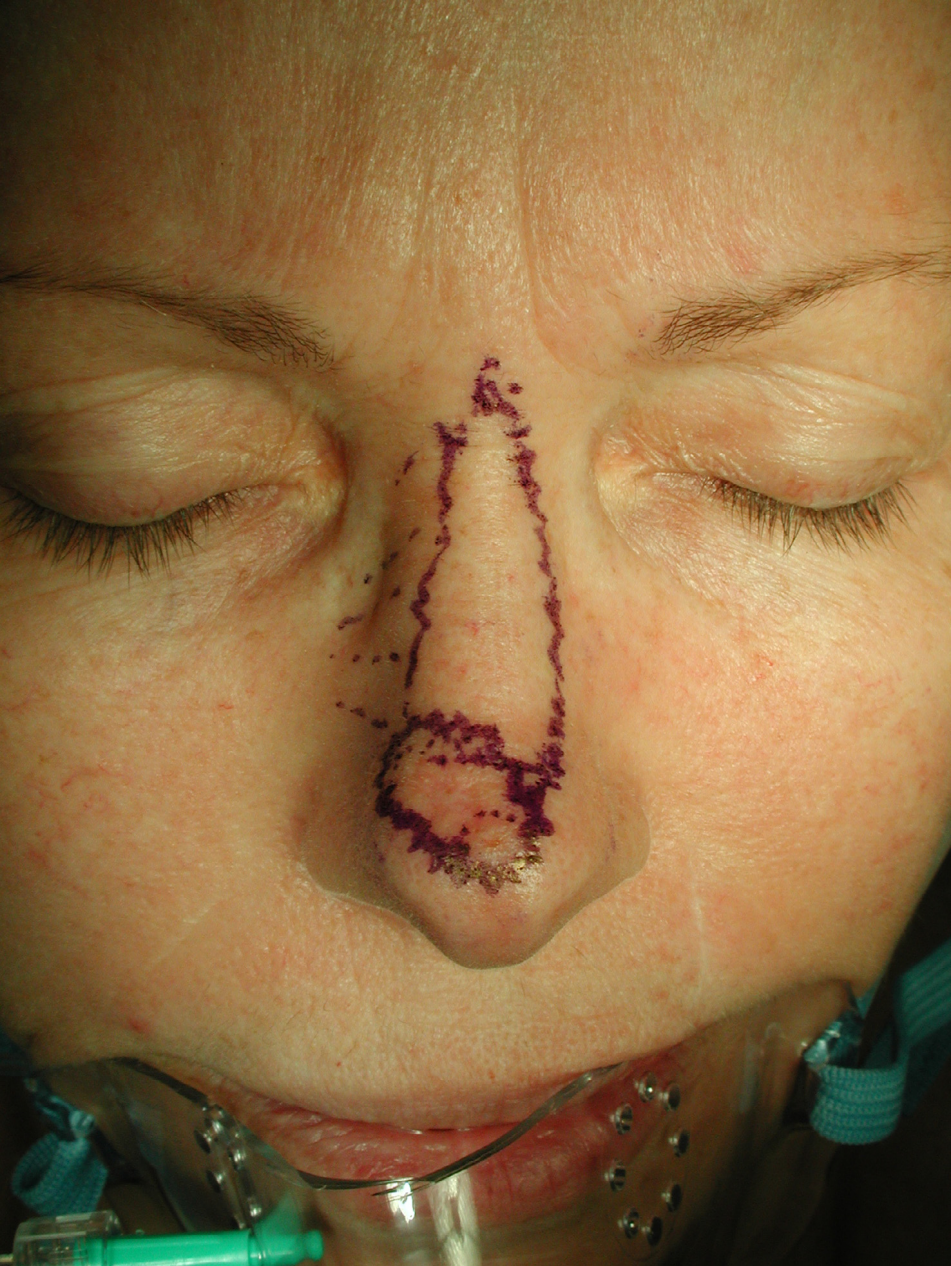
The lesion at nasal tip and the flap design.

Between January 2010 and June 2020, the modified horn flap was performed in over 60 cases by the senior author (R.A.) for nasal tip reconstruction of defects after excision of primary skin cancers. Mean patient age was 63. The defect size ranged from 1 to 2 cm vertically and from 1.5 to 3 cm horizontally. Among all patients 20% had an extension of the skin cancer to the ala nasi. The surgical technique includes excision of the tumor with adequate tumor resection margins. The flap is designed as an island horn-shaped flap whose inferior border is the superior border of the defect and superior border is the glabella (Figure 1). All incisions are made in a “deckling”^4^ fashion with a 15° Beaver Blade to avoid pin-cushion deformity. Flap incisions begin superiorly along each side of the horn flap subcutaneously as far as the nasalis muscle. The superior alar artery^5^ is carefully identified 1 to 3 mm above the root of the alar groove and preserved with the adjacent caudal half (or more) of the nasalis muscle. Adjacent soft tissue is dissected off the artery and nasalis muscle laterally for 15–20 mm, under loupes. On the side contralateral to the artery, an incision is made along the nasalis muscle through to the nasal cartilage, lifting the muscle and deep veins within the flap towards the pedicle side. Flimsy deep veins are included for additional drainage of the flap, with continued submuscular dissection further laterally as required. The flap is then advanced into the defect with minimal tension on the nasalis and superior alar artery (Figure 3).

Routine incorporation of the superior alar artery within the flap followed partial skin necrosis (6 mm diameter) in a conventional horn flap in 2010. There have been no flap losses since then.

## Discussion

The contribution of the superior alar artery forming the vascular axis of the flap plays a vital role in flap survival together with the nasalis muscle. It is based on a horn flap concept as originally described by O'Donnell et al.^2^ with the additional blood supply of the superior alar artery for more reliable circulation. This also allows significant modification of the shape of the horn, which maybe curved in either direction, or straight, even if the superior blood supply (via nasalis/procerus) is compromised or sub-totally severed. The flaps described by Marchac and Tothet al.^4^ and de Fontaine et al.^5^ depict partial skin attachment and have a more superiorly based blood supply. Furthermore, these flaps were wider and extended to the nose cheek area. The “deckling”^6^ incisions are finely irregular serpentine skin incisions similar to a jigsaw puzzle, or a compact irregular sine wave. The W-plasty as described by Borges^7^ together with variations of w-plasty for the areolar inset^8^ during breast reduction or mastopexy are certainly useful. They are better than linear incisions but occasionally leave alternate strokes of each “W” visible – or visible apices of the W. In our experience, the “deckling”^6^ incisions are particularly effective for the nose and the cosmetic advantages are greater on the face generally, compared to those seen on other parts of the body.

## Conclusion

**Figure 2:**
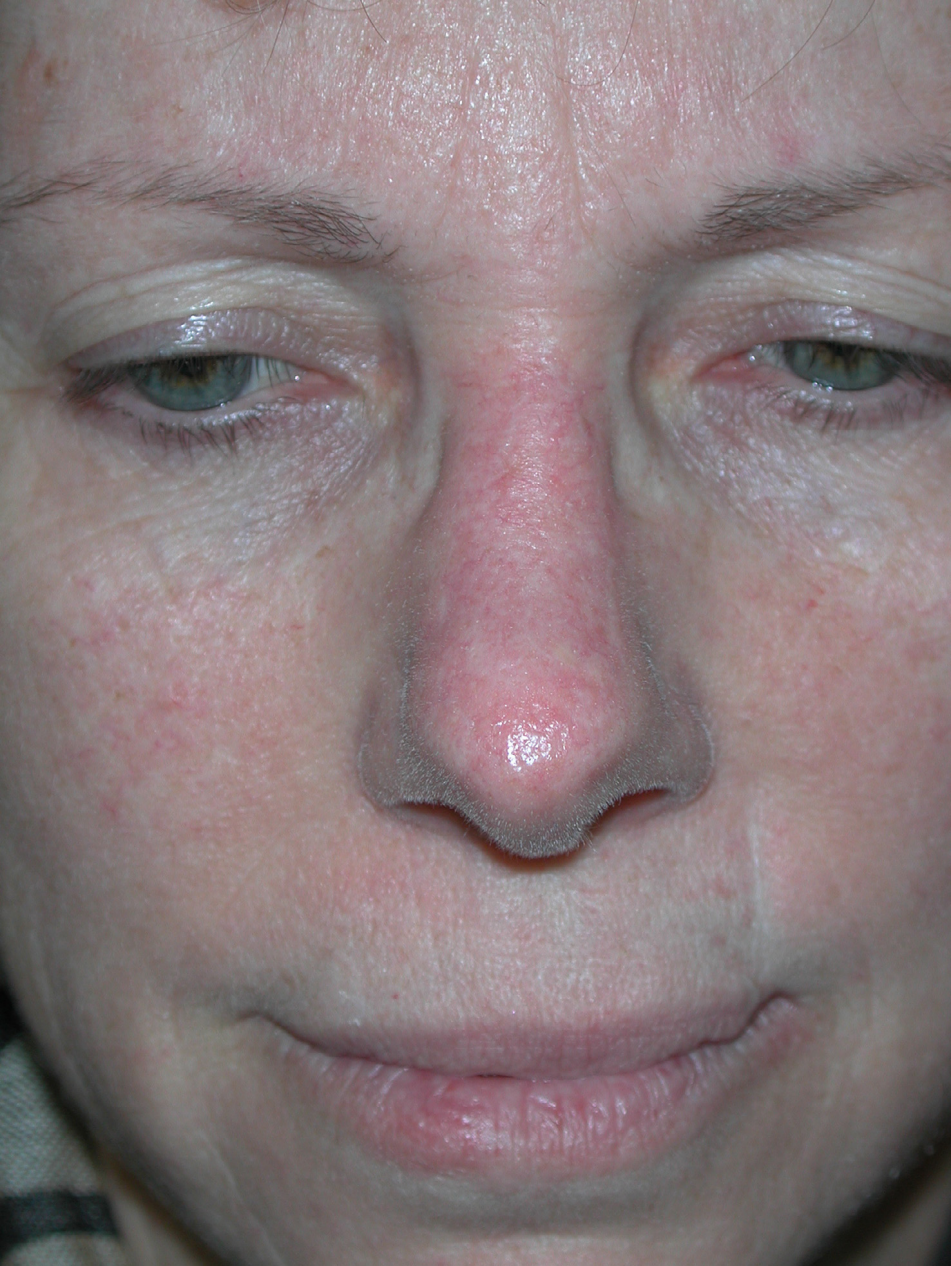
The postoperative result in the same patient after 3 months.

The modified horn flap is a reliable and versatile island flap for the reconstruction of the nasal tip in a one-stage operation. The “deckling”^6^ incisions, the contribution of the superior alar artery with the nasalis muscle, together with the fact that the flap is generated from the nose (excellent color and contour match), provide successful functional and aesthetic results (Figure 2)

**Figure 3:**
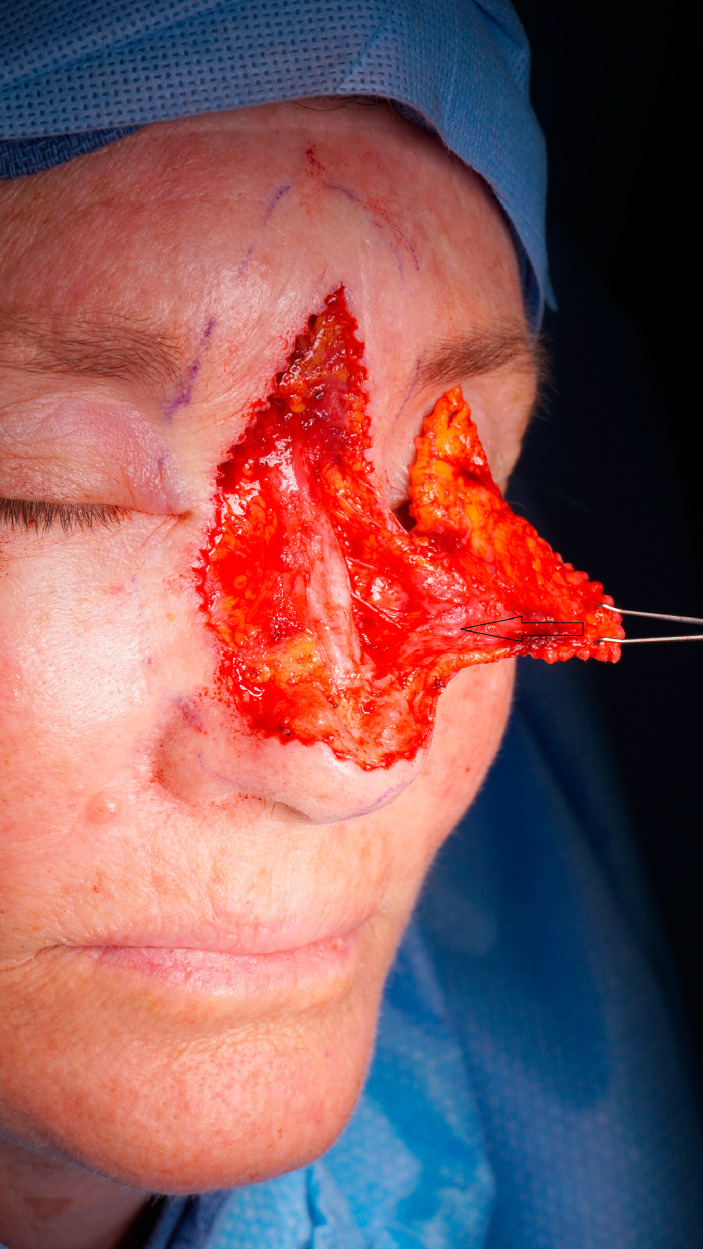
The superior alar artery at the lower border of nasalis muscle.

## Declaration of Competing Interest

The authors declare no conflict of interest
